# Supplementary material for: School well-being in primary school children with chronic illness. A prospective cohort study
Source: PeerJ. 2024 Nov 18;12:e18280. doi: 10.7717/peerj.18280 (PMC11583910; doi:10.7717/peerj.18280)
Supplement: Supplemental Information 1 [file peerj-12-18280-s001.docx]

| **Variable** | **Concept** |
| --- | --- |
| idklasse | Class ID |
| schulid | School ID |
| EFB1_cshcn | SHCN status (no/yes) end of Kindergarten |
| EFB3_cshcn | SHCN status (no/yes) end of Grade 1 |
| cshcn.all | SHCN status (no/yes) end of Kindergarten or Grade 1 |
| hinw.all | Any school-related chronic health condition (no/yes) |
| CE3groups | 3-level variable indicating SHCN, any school-related chronic health condition (but no SHCN), or no SHCN/no chronic health condition |
| Geschl | Sex |
| mig | Immigrant status |
| alleinerz | Single parent family |
| age.mu | Mother’s age at school entry of child |
| age.va | Father’s age at school entry of child |
| windex.sum | Socio-economic status (Winkler’s Index) |
| Mehrling | Multiple at birth |
| stillen | Breast-feeding |
| Town | Area of residence (rural/urban) |
| GesBew | Completion of recommended well-child visits |
| CEFam | Chronic disease in the family (sibling or parents) |
| KFB_sozInt_t | SWB: Social Integration (t value) |
| KFB_lernfreude_t | SWB: Joy of Learning (t value) |
| KFB_selbstkonzept_t | SWB: School Self-Concept (t value) |
| multimpinstance | Multiple imputation instance |
